# A study on the impact of the users’ characteristics on the performance of wearable fall detection systems

**DOI:** 10.1038/s41598-021-02537-z

**Published:** 2021-11-26

**Authors:** José Antonio Santoyo-Ramón, Eduardo Casilari-Pérez, José Manuel Cano-García

**Affiliations:** 1 Departamento de Tecnología Electrónica, Universidad de Málaga, CEI Andalucía TECH, E.T.S.I. Telecomunicación, Bulevar Louis Pasteur 35, 29010 Málaga, Spain; 2 Departamento de Tecnología Electrónica, Instituto Universitario de Investigación en Telecomunicación (TELMA), Universidad de Málaga, CEI Andalucía TECH, E.T.S.I. Telecomunicación, Bulevar Louis Pasteur 35, 29010 Málaga, Spain

**Keywords:** Electrical and electronic engineering, Biomedical engineering, Information technology, Scientific data

## Abstract

Wearable Fall Detection Systems (FDSs) have gained much research interest during last decade. In this regard, Machine Learning (ML) classifiers have shown great efficiency in discriminating falls and conventional movements or Activities of Daily Living (ADLs) based on the analysis of the signals captured by transportable inertial sensors. Due to the intrinsic difficulties of training and testing this type of detectors in realistic scenarios and with their target audience (older adults), FDSs are normally benchmarked against a predefined set of ADLs and emulated falls executed by volunteers in a controlled environment. In most studies, however, samples from the same experimental subjects are used to both train and evaluate the FDSs. In this work, we investigate the performance of ML-based FDS systems when the test subjects have physical characteristics (weight, height, body mass index, age, gender) different from those of the users considered for the test phase. The results seem to point out that certain divergences (weight, height) of the users of both subsets (training ad test) may hamper the effectiveness of the classifiers (a reduction of up 20% in sensitivity and of up to 5% in specificity is reported). However, it is shown that the typology of the activities included in these subgroups has much greater relevance for the discrimination capability of the classifiers (with specificity losses of up to 95% if the activity types for training and testing strongly diverge).

## Introduction

Falls among elderly are a cause of major concern for health systems. According to the World Health Organization (WHO)^1^, around 646,000 mortal falls occur each year in the world, while 37.3 million falls are severe enough to require medical attention. Most of these accidents are suffered by adults older than 65 years. Only in USA, 27.5% of adults aged over 65 years reported at least one fall in 2018^2^. As a result of falls, 5 to 11% of older individuals experience serious injuries, including hip fractures, subdural hematomas or/and severe tissue or head injuries^3^. A long lie on the floor after a fall without receiving medical assistance is directly associated to a dramatic increase of the mortality rate, hospitalization and care home admissions^4^. In this context, it is not surprising that fall detection systems (FDSs) built on wearable devices have attracted great interest from the scientific community in the field of telemedicine and remote monitoring of biosignals.

In contrast with context-aware and video-based alternatives, systems based on wearables may benefit from the widespread popularity and low-cost of these devices, while providing an almost-ubiquitous tracking that does not interfere with the privacy of the final users. One key and controversial aspect in the development of a wearable FDS is the procedure followed for its evaluation^5^. Due to the understandable and inherent difficulties of testing these detectors in a real environment with older patients, fall detection algorithms are typically gauged with movements generated by a group of experimental subjects.

FDSs are binary pattern recognition systems, trained or designed to discriminate activities of daily living (ADL) from violent or agitated movements that can be identified as falls. Although they can also include other types of sensors (such as barometers, magnetometers or heart rate monitors), almost all wearable FDSs proposed in the literature base their detection decision on the analysis of the signals captured by accelerometers and, in some cases, gyroscopes, which are commonly integrated in the same IMU (Inertial Measurement Unit). In this way, the datasets used for the evaluation of the FDSs are generated by conducting a series of experiments, in which a group of volunteers execute a predefined and structured set of ADLs (climbing stairs, walking, running, lying down, etc.) and various types of falls (trips, slips, crashes, collapses, etc.) while transporting an IMU in one or more body locations.

Due to the research interest in wearable FDSs, numerous repositories have been released in recent years (see, for example, the study in^6^ for a full review and comparison of these datasets). These datasets offer an appealing global benchmarking tool that is increasingly being used by specialists to assess the efficacy of the proposals for new detection algorithms. In a significant number of cases, the documentation of these datasets habitually provides the basic data (mainly age, gender, height and weight) of the subjects that participated in the experiments. However, as it is pointed out in the review presented by Ren and Peng in^7^, most evaluation frameworks of FDSs do not consider the importance of the users’ characteristics on the results obtained by the movement classifiers. In fact, there are not clear guidelines or criteria to systematically select the number and typology of the participants employed to parameterize and evaluate the FDSs (typically implemented on machine learning models). This aspect is of special relevance if we consider that the detectors are not expected to be adjusted (or trained) with movements, especially falls, of the final users (older people) for whom these devices are targeted. To cover this lack, this paper focuses on analyzing the impact of the physical characteristics of the experimental users on the accuracy of the FDSs. The general goal is to identify those characteristics with a greater influence, aimed at defining recommendations on the typology of the subjects that should be part of the databases with which detection algorithms are parameterized or trained.

The paper is organized as follows: “Related works” section reviews the related literature. “Methods: definition of the evaluation framework” section describes the evaluation framework used to analyze the effect of the characteristics of the participants while “Results and discussion” section presents and discusses the results achieved when this analysis framework is applied to different existing repositories. To conclude, “Conclusions” section summarizes the main conclusions of the work.

## Related works

FDSs can be envisaged as a special case of Human Activity Recognition (HAR) system. In this regard, Lockhart and Weiss distinguished in^8^ two general types of models (which can be hybridized) to build a HAR system based on machine learning:

- Impersonal universal models, which employ training data from a set of users that will not test the model. The main advantage of this model type is that it is built once, so that no labelled data or extra training phases are required from the target users.
- Personal models, which only consider training data from the final (test) user. This personalization is achieved at the cost of disturbing the target user (the patient to be monitored) to obtain the corresponding labelled data. In the case of FDSs, for obvious reasons, actual fall samples from the elderly patients to be monitored can be extremely difficult to obtain.

The results presented by the same authors in^9,10^ highlighted the importance of personalizing the training sets with which the machine learning methods are trained in smartphone-based HAR systems, as they clearly outperform ‘impersonal’ models (trained with a different panel of subjects). Likewise, Cvetković et al. showed in^11^ that the accuracy of a HAR system (aimed at distinguishing up to eight diverse movements, including falls) strongly degrades (from 86 to 73%) when it is tested with a person with a different height from that of the subjects included in the training set. To reduce the problem, authors scale the data that feed the classifier by multiplying the input features by the ratio between the height of the test user and the average height of the users in the training set.

Following this line of thinking, Saeb et al. have also underlined the need of validating the decisions of classifiers based on data captured by wearables with the target population for whom the final application is intended. In particular, the bibliographical analysis presented by these authors in^12^ uncovers that most studies on machine learning algorithms aimed at clinical prediction and diagnosis are evaluated through a ‘record wise’ strategy (instead of ‘subject-wise’ policy), that is to say, by using training and test datasets that do not take into account the actual population expected for the clinical use of the classifier. Authors demonstrate that this strategy, which does not contemplate the use of newly recruited individuals for the testing phase, tends to massively produce optimistic predictions of the efficacy of wearable-based classifiers in real clinical use-cases.

The ‘personalization’ of certain basic wearables is not uncommon. For example, during the setup process, pedometers demand users to input height and gender to estimate the step/stride length. In fact, recordings from a single inertial sensor can be employed to deduce the gender, age and height of a user^13^. In this respect, Masuda and Maekawa have also shown that user characteristics (gender, height, weight, dominant hand) can be estimated with machine learning strategies uniquely from basic activities such as washing dishes or walking^14^.

As it refers to FDSs, interpersonal differences may hinder the accuracy of fall detectors as long as personal characteristics may be important to determine several aspects in the dynamics of a falls and ADLs.

Shen et al. developed a smartphone-based FDS in^15^ and confirmed that body movements (including falls) tend to vary according to the difference in height, weight, and gender of the experimental users. For example, if the subject falls while standing, the user’s height may result a key element in the change of the position of the head. Similarly, the duration and nature of the free-fall period as well as the acceleration peak caused by the impact against the floor may strongly rely on the user’s height and weight. Besides, weight (obesity, thinness, …) can also influence the ergonomics of the user and the position in which the sensor can be transported in the most comfortable way (e.g. on the chest). Similarly, some works on FDSs have observed that acceleration measurements or points of impact may be strongly affected by users’ weight or height^16–18^. Ando et al. even suggested in^19^ that as long as the acceleration signal is strictly correlated with the user characteristics (height and weight), signals collected by the accelerometer have to be normalized before being inputted to the classifiers. These authors argue that this normalization can make the system insensitive against the user’s characteristics. In contrast, Kaenampornpan et al. state in^20^ that the user's height does not significantly affect detection criterium (the difference between the values of the acceleration components before the activity starts and when the activity stops), at least for a certain groups of ADLs. No numerical evidences of this statement are provided.

In the interesting work by Shawen et al. in^21^, authors evaluate the effectiveness of four fall detection methods (based on the measurements of a smartphone and machine learning classifiers) when they are applied to the movements of individuals with lower limb amputations. Results evince that the performance of a detector trained on control non-amputee volunteers was not statistically different from that those achieved with a model trained on amputee population.

Gender is other circumstance that must be contemplated as it also impacts on the recurrence and consequences of falls. Among older adults, non-fatal fall related injuries disproportionately affect women^22^. Anatomic differences between genders may be a focal factor to select the best position and orientation of the wearable and even the style with which certain individual movements are executed^23^.

Another key debate about FDSs is focused on the appropriateness of the results and configuration of FDSs trained with intentional falls mimicked by young and healthy subjects when they are extrapolated to real monitoring scenarios with older adults. Seniors have longer reaction time when compared to younger individuals. There is approximately a 25% increase in simple reaction time from the twenties to the sixties, with further significant slowing beyond this age^24^. In this respect, Klenk et al. found significant differences between the inertial measurements captured during actual backward falls of four older persons and falls simulated by young volunteers^25^. On the other hand, Kangas et al. showed in^26^ that a wearable waist-worn accelerometer based FDS trained with falls simulated by 20 middle-aged (40–65 years old) experimental subjects can be effective to avoid false alarms when it is tested against the ADLs captured from 21 older people (aged 58–90).

In 1994, O’Neill et al. interviewed 1243 subjects aged over 50 to evaluate the importance of gender and age in the characteristics of falls experienced in the previous 4 months^27^. The study showed that age and sex differences may cause a clinically significant variation in the typology of falls. For example, males aged 50–64 were reported to be more likely to fall sideways and less likely to fall forwards than older males. Moreover, falls may be affected by a myriad of other personal factors. Epidemiological studies have revealed that sporting habits, vision, and subjective fall risk are key factors associated with fall recurrence. Age may even impact on the environment where most falls occur. So, the proportion of women who fell inside the home increases with age^28^.

In the related literature, the design of systems for fall detection has not been completely oblivious to this need of customizing the detectors. In this regard, strategies for fall detection criteria can be roughly classified into two basic types^29–31^, depending on weather they rely on a Threshold-Based (TB) or a Machine Learning (ML) algorithm. TB architectures trigger a fall alarm as soon as one or several parameters directly derived from the inertial sensors (e.g. the acceleration magnitude) exceed or fall below a certain preset value. Contrariwise, ML algorithms offer a more sophisticated and flexible solution to the problem of pattern recognition and avoid the need of establishing an arbitrary and rigid threshold (or set of thresholding values) to presume the occurrence of a fall. Under a supervised scheme, a FDS based on a ML classifier is trained with a dataset of labelled samples containing features computed from the measurements of the wearables and representing the two types of movements (falls and ADLs) that must be discriminated. During the training process, the ML algorithm is capable to self-configure to maximize the success ratio of the binary output decision. In the case of deep-learning architectures^32^, the model may even be directly inputted by the collected measurements, so that the method is able to extract the most convenient features to optimize the results without requiring a ‘manually-engineered’ selection of inputs. Due to the complex nature of human movements (and in particular, those caused by fall accidents), diverse studies^29,33^ have shown that ML methods generally outperform the behavior of the TB approximations.

In the literature on threshold-based fall detectors, there are a certain number of works in which this detection threshold is tuned or defined according to the personal characteristics of the subject to be monitored. For example, in^34^, Cao et al. presents a smartphone-based FDS that analyzes the accelerometer signals using a sliding semi-overlapping time widow and two acceleration thresholds. The window-size for the signal analysis and threshold are selected depending on the sex, age and BMI (Body Mass Index) of the subject. A similar approach is followed by Rungnapakan et al. in^35^ as the threshold values to detect the impact force of the falls were set separately for each weight range of the experimental users. Wu and Tsai present in^36^ another smartphone-based FDS using a threshold-based algorithm in which the threshold level is tuned and ‘heuristically’ adapted for each user. Age, weight, height and level of activity of the user are considered into the equation that computes the decision threshold in the SP-based FDS presented by Sposaro in^37^. Wu and Tsai propose in^36^ an adaptive threshold based on user’s gender to detect falls based on the energy of the movements calculated from the DCT (Discrete Cosine Transform) of the acceleration magnitude. In all these works, authors do not evaluate the advantages (e.g. in terms of the accuracy, sensitivity or specificity of the FDS) of tuning and particularizing the parameters of the detectors depending on the user’s characteristics with respect to the case in which a generic threshold is defined.

After analyzing other threshold-based FDS, Wu et al. underlined in^38^ the importance of a proper selection of the threshold value to discriminate falling from other conventional activities that imply a sudden rotation of the body (such as lying). These authors claim that the robustness and reliability of their proposal could improve if the value of the threshold could be derived from the study of datasets captured from subjects with different age and gender. In this vein, Ren et al. showed in^39^ that the magnitude in the acceleration during for types of ADLs and falls are dependent on the user’s gender and age. In particular, stronger peaks were proved to be related to young and male subjects. Thus, these authors propose to combine an at-group and a light-self-tuning strategy to set the threshold. At-group strategy considers different age and gender partitions to estimate the best threshold for each group using ADL movements. This group threshold is then optimized and fine-tuned to provide an individual threshold for each experimental subject.

In any case, thresholding methods are in general too rigid to adapt to the huge variability of human movements and, in most testbeds, they normally produce a non-negligible number of false positive (false alarms) and false negatives (unnoticed falls). As aforementioned, ML and deep learning methods usually exhibit a much greater adaptability to identify human dynamics^40^. However, the almost automatic self-configuration of these classifiers hinders any ‘manual’ tuning of their hyperparameters based on the subject’s characteristics. Abbate et al. state in^41^ that FDSs should be capable of self-learning to adjust their parameters to the characteristics of the particular user who is wearing it but they do not provide any concrete solution to implement this guideline. Aziz et al. in^33^ or Tomkun et al. in^42^ also suggested taking into consideration subject anthropometric information (height, mass, age, etc.) to train or tailor machine learning detectors (such as SVM) but just as a future work. In particular, Tomkun proposed generating extensive datasets to parameterize specific SVM-based detectors depending on user’s height, weight and gender^42^. Nevertheless, user’s characteristics have not been considered as input features in any ML-based detector in the related literature.

In short, most studies on wearable FDS based on artificial intelligence techniques neither evaluate nor consider the possible impact of user characteristics on the configuration or performance of the detection algorithms. In the next section, we analyze the generalizability of the learning of ML detectors and their capability to extrapolate results when training and test users have different physical characteristics.

## Methods: definition of the evaluation framework

### Selection of data repositories

Currently, there are more than 25 public repositories available for the off-line analysis of wearable FDSs^6^. As previously mentioned, these repositories basically consist of numerical time series representing the measurements of inertial sensors located on one or several positions of the body. The measurements are almost unanimously captured from the movements of a set of experimental subjects, who are instructed to execute a variety of ADLs and mimicked falls. The time series are labelled in the databases according to this binary classification of the movements.

Many works^43–47^ have suggested that the optimal position of an inertial sensor for a fall detector is waist (or falling that, the chest) as it is close to the center of gravity of the human body. Thus, aiming at comparing the different classifiers on an equal basis, we focus on the 14 existing datasets that include measurements collected at the waist. For the study, we discard those repositories that do not inform about the particular characteristics of the volunteer that executed each movement (although in most cases the average values of these characteristics of the participants are provided). In addition, we do not consider those datasets that contain less than 400 movement samples since partitioning the traces of those repositories into different groups according to the individual characteristics of the subjects could result in biased subsets with insufficient and unrepresentative samples. After screening the available databases with these criteria, we selected five datasets (DOFDA, Erciyes, SisFall, UMAFall and UP-Fall), whose main characteristics are presented in Tables 1 and 2.

**Table 1:** Basic data of the repositories employed for the analysis.

Dataset	Source	Number of types of ADLs/falls	Number of samples (ADLs/falls)	Duration of the samples (s)	Sampling rate (Hz)	Accelerometer range (g)
DOFDA	^62^	5/13	432 (120/312)	[1.96–17.262] s	33	± 16
Erciyes University	^63^	16/20	3302 (1476/1826)	[8.36–37.76] s	25	± 16
SisFall	^54^	19/15	4505 (2707/1798)	[9.99–179.99] s	200	± 16
UMAFall	^64^	12/3	746 (538/208)	15 s (all samples)	20	± 16
UP-Fall	^65^	6/5	559 (304/255)	[9.409–59.979] s	14	± 8

**Table 2:** Characteristics of the experimental subjects in the employed datasets.

Dataset	Number of subjects (females/males)	Age (years)	Weight (kg)	Height (cm)
DOFDA	8 (2/6)	[22–29]	[60–94]	[173–187]
Erciyes University	17 (7/10)	[19–27]	[47–92]	[157–184]
SisFall	38 (19/19)	[19–75]	[41.5–102]	[149–183]
UMAFall	19 (8/11)	[18–68]	[50–97]	[156–193]
UP-Fall	17 (8/9)	[18–24]	[53–99]	[157–175]

The benefits of combining the analysis of the accelerometry-signals with those measured by other inertial sensors (in particular the gyroscope) are still under discussion^48,49^. So, although some of these traces also include data obtained from a gyroscope and/or a magnetometer, for the sake of simplicity, we concentrate our research on the accelerometer measurements provided by abovementioned datasets.

### Selection of data classifiers

The core of a wearable FDS is the decision algorithm that discriminates falls from ADLs. As already stated, due to the simplistic approximation of TB methods to the complex dynamics associated to falls, they are generally outperformed by ML strategies^33^. For this reason, for our analysis we select four well-known supervised machine learning algorithms, which are commonly employed in the related literature^50–52^:

- Support Vector Machine (SVM). To construct the model, we alternatively consider four possible popular kernels to transform the data: linear, cubic, quadratic and medium gaussian.
- K-Nearest Neighbors (K-NN). In this case, we check the performance when four different functions (Euclidean, Minkowski, Chebychev and cosine) are used to compute the distance to the nearest neighbors. Additionally, we consider three values for K (5, 10 & 50).
- Naïve Bayes (NB). We contemplate two cases for this classifier depending on the procedure to approximate the distribution of the input data: Gaussian function and KDE (Kernel Density Estimation).
- Decision Tree (DT). To define the topology and dimension of the decision tree, we tested two different policies. In the first case, following a ‘coarse’ approximation, a simple structure of up to four splits is allowed. Under the second (‘fine’) policy, the limit to the number of splits is set to 100 to enable a more flexible tree topology. In both cases the Gini's diversity index was adopted as the splitting criterion.

All the models (totaling 30 variants: 4 for SVM, 12 for KNN, 2 for NB and 2 for DT), were hyperparameterized and implemented (trained and tested) with Matlab (version 9.9 -R2020b-) scripts using the Statistics and Machine Learning Toolbox^53^. For all the secondary hyperparameters not commented here (e.g. procedure for objective-function minimization in SVM, distance weighting function for k-NN, etc.) we used the default values provided by the corresponding scripts in the toolbox.

### Selection of input features

A key element for the performance of a machine learning classifier is the selection of the input features which describe the data. As already mentioned, our study will focus on the values captured by the tri-axial accelerometers ${(A}_{{x}_{i}},{\text{A}}_{{\text{y}}_{\text{i}}} \; \text{ and } \; {A}_{{z}_{i}}$ for the *i*-th measurement), which are provided by the repositories. From these components of the acceleration, we can straightforwardly compute the signal magnitude vector (*SMV*):

1 $${SMV}_{i}=\sqrt{{A}_{{x}_{i}}^{2}+{A}_{{y}_{i}}^{2}+{A}_{{z}_{i}}^{2}}$$

Movement samples existing in the datasets present a variable duration. However, as an impact against the floor provokes a sudden increase of the acceleration, every movement in the traces will be characterized only from the measurements taken during a time interval of 1 s just before and after the highest detected acceleration peak, which is he period in which the fall is most likely to have occurred. Thus, the input features will be derived from this time observation window of 2 s, while the rest of the time series of each sample will be ignored by the detection algorithm.

In a previous work^6^, we defined and analyzed 12 statistical acceleration-based features that have been proposed and studied by different works in the literature, as they can describe different properties of the dynamics of the human body during a fall. These feature, which are computed from the acceleration components during the observation window, are defined the following (refer to^6^ for a more detailed formal description):

- The mean value of *SMV*, which informs about idea the average mobility suffered by the body.
- The standard deviation of *SMV*, which characterizes the variability of the acceleration.
- The mean absolute difference between two consecutive values of *SMV*, which can offer an indication of a sudden fluctuation of the acceleration caused by a fall.
- The mean rotation angle, which may describe any possible alteration of the body orientation of the body.
- The mean magnitude of the vector formed by the two acceleration components that are parallel to the floor plane (which informs abut changes in the verticality of the body).
- The magnitude of the maximum difference between any two acceleration vectors (within the observation interval), which also offers a measurement of the variability of the acceleration components.
- The maximum value of *SMV*, as a direct descriptor of any possible impact against the floor.
- The minimum of the *SMV*, which can characterize the ‘valley’ or brusque decay of the acceleration magnitude during the free-fall phase preceding the impact of a fall.
- The skewness of third-central moment of the series *SMV*_*i*_, which informs about the asymmetry of the acceleration distribution function.
- The Signal Magnitude Area (*SMA*)^54^, which is a feature commonly considered to assess the physical activity.
- The sum of the energy estimated from the Discrete Fourier Transform of the acceleration components in the three axes during the observation interval^55^, which may be used to detect rapid body movements.
- Mean of the autocorrelation function of *SMV*, as falls movements are supposed to be less correlated than ADLs, which normally present smoother and slower alterations of the acceleration.

After massive grid search with combinations of the different 12 features and the corresponding Analysis of Variance (refer to our previous works in^56,57^ for a full description of the method), we conclude that the best behavior of the four classifiers and for almost all the combinations of considered hyperparameters is achieved when all the features are simultaneously considered. Techniques for dimensionality reduction of these features, such as PCA (Principal Component Analysis) or mRMR (Minimum Redundancy Maximum Relevance), were employed, although no significant improvements were obtained when the derived features were inputted into the classifiers. Consequently, we utilize this set of 12 features, which present an evident physical interpretation, as a first alternative to dimension, train and test the detection algorithms.

In any case, as the selection of these 12 features may still seem arbitrary, we also consider, as an alternative set of features to feed the algorithms, those extracted by the *hctsa* (highly comparative time-series analysis) MATLAB software package^58^. *hctsa* library analyzes thousands (7700) of candidate features from univariate time-series to produce a low-dimensional representation of the data^59^. In our case, this tool was programmed to cluster the data into 12 groups in order to also select the 12 most representative features from the series of the acceleration SMV computed during the observation window of the samples in each dataset. From some preliminary tests (not shown here) we concluded that no significant gain is achieved in the classifiers if a higher number of *hctsa* features is considered.

With independence of the set of features employed to train the detectors, before being split into train and test sets and inputted to the detector for training and testing, the feature data were standardized by using a standard or z-score normalization.

### Selection of performance metrics and train-test split strategy

In order to evaluate the trained detectors as binary classifiers, we estimated the sensitivity (*Se*) or recall, defined as the proportion of falls -or positives- in the test subset that are correctly identified, and specificity (*Sp*), as the ratio of ADLs which are not misinterpreted as falls so that they do not trigger a false alarm. In contrast with other measures of test accuracy (such as the accuracy or F1 score), these metrics are not skewed if the number of existing ADLs and falls in the datasets are unbalanced (as is the case of the datasets employed in this study). In addition, in order to describe the trade-off between these two statistical measures through a single variable, we also computed the geometric mean of *Se* and *Sp* ($\sqrt{Se\cdot Sp}$) as a global performance metric.

To calculate these quality metrics, we applied a fivefold cross validation procedure in which the datasets were split into five partitions of the same length. Every model of the classifier was then trained and tested five times. In each iteration, a different fold was employed for testing while the other four partitions were merged to form the training set (following the typical 80–20 rule). Results obtained during the test phase of each iteration are then averaged to generate the final metrics.

## Results and discussion

The evaluation framework described in the previous section is now utilized to assess the impact of the characteristics of the subjects used to train the ML algorithms on the discrimination capacity of the detector.

### Results for a fair distribution of samples

Aiming at establishing a baseline reference, we firstly evaluate the performance of all the ML classifiers (and their different configurations) for the ideal or ‘fair’ case in which the subsets used for both training and testing contain samples of all the experimental subjects. In particular, the partition of the datasets was randomly generated although it was guaranteed that samples of all the types of activities (ADLs and falls) and all the experimental subjects were included in the five folds.

Averaged results for the five testing folds of this ‘reference case’ are presented in Table 3. To characterize the degree of uncertainty of the results, the last column of the table incorporates the standard deviation of the global performance metric ($\sqrt{{{S}}{{e}} \cdot {{S}}{{p}}}$), computed from the five experiments executed during the fivefold cross-validation of the models. For the sake of simplicity, the table only shows the results of the four variants of the classifiers that achieved the best global performance metric. The best results and classifier (marked in bold) will be utilized as a reference in the analysis in the following sub-sections.

**Table 3:** Performance metrics of the four best performing classifiers using fivefold cross validation and a ‘**fair**’ distribution of the samples between the training and testing subsets.

Dataset	Features	Algorithm and hyperparameters	Se (%)	Sp (%)	$\sqrt{Se\cdot Sp}$^a^
DOFDA	**HCTSA**	**Naive Bayes (Gaussian)**	**97.37**	**100.00**	**98.67** ± 1.27%
	HCTSA	SVM (linear kernel)	99.01	98.33	98.65 ± 1.77%
	Own selection	KNN (Euclidean, 10 neighbors)	98.02	98.18	98.08 ± 2.08%
	HCTSA	SVM (quadratic kernel)	99.34	96.67	97.97 ± 2.44%
Erciyes	**Own selection**	**SVM (quadratic kernel)**	**99.62**	**99.18**	**99.40** ± 0.17%
	Own selection	SVM (medium gaussian kernel)	99.34	98.98	99.16 ± 0.17%
	Own selection	KNN (cosine, 5 neighbors)	99.07	99.05	99.06 ± 0.12%
	Own selection	KNN (Minkowski, 5 neighbors)	99.45	98.64	99.04 ± 0.12%
SisFall	**HCTSA**	**SVM (cubic kernel)**	**99.78**	**99.96**	**99.87** ± 0.13%
	HCTSA	SVM (quadratic kernel)	99.78	99.96	99.87 ± 0.19%
	HCTSA	SVM (medium gaussian kernel)	99.11	99.96	99.54 ± 0.12%
	HCTSA	DT (Fine)	98.89	99.96	99.42 ± 0.23%
UMAFall	**Own selection**	**KNN (Euclidean, 10 neighbors)**	**98.93**	**98.73**	**98.83** ± 0.86%
	Own selection	DT (Coarse model)	98.38	98.99	98.67 ± 1.93%
	Own selection	SVM (medium gaussian kernel)	97.87	99.24	98.55 ± 0.55%
	Own selection	KNN (Euclidean, 5 neighbors)	98.93	97.97	98.45 ± 1.10%
UP-Fall	Own selection	**SVM** (linear kernel)	99.59	98.02	98.80 ± 1.31%
	Own selection	SVM (medium gaussian kernel)	98.78	98.82	98.79 ± 1.32%
	Own selection	KNN (Euclidean, 10 neighbors)	99.18	97.23	98.20 ± 1.31%
	Own selection	KNN (Euclidean, 5 neighbors)	98.78	97.62	98.19 ± 1.65%

^a^The last column includes the standard deviation of the measurement of $\sqrt{Se\cdot Sp}$ for the five-fold tests.

As can be seen, in general, SVM (under different configurations of the kernels) and, to a lesser extent, KNN, are the algorithms that offer the best classification of the patterns. This conclusion is consistent with most of the comparative analyses carried out in the literature that have compared the performance of ML classifiers in FDSs (see, for example, the studies in^52,60,61^). Likewise, the results show that the selection of the 12 characteristics (‘own selection’) commented in “Selection of input features” section  (which have a clear physical interpretation in the characterization of the activities) can even lead to a better behavior than the choice of 12 features based on the massive test of ‘abstract’ statistical features offered by the *hctsa* tool. Nonetheless, there are combinations (datasets and ML models) for which the best behavior is achieved with *hctsa*-derived features. Further studies should thoroughly investigate if the use of this type of tools for the automatic selection of features can be helpful in the design of fall detection algorithms.

In any case, the evaluation indicates that the ML algorithms are capable of producing an acceptable recognition rate, with sensitivities and specificities (for the methods presented in Table 3) always higher than 97%.

### Results for a distribution of samples based on personal characteristics

To assess the impact of the subject’s characteristics on the discrimination capacity of the classifiers, we repeated the previous analysis by modifying the composition of the subsets used for training and testing. Now, for all the cases, we included all the samples captured from the same user just in one the subsets (training or testing). Thus, the models are always tested with traces obtained from users different from those who generated the training subset. This could correspond to a realistic application scenario of an FDS in which the target user (e.g. a particular elderly person) does not directly participate in the training of the fall detector.

As the selection criterion to cluster the users into the testing or training group, we utilize one of the following five characteristics: weight, height, BMI or Body Mass Index (computed as the subject's weight in kilograms divided by his/her squared height in meters), age and gender.

For the first four characteristics, which are defined by a specific numerical value, the training set included the samples of 80% of the subjects, while the remaining 20% were used in the test phase. In this way, the distribution of the samples between the training and testing sets (by roughly following the 80/20 rule) was similar to that used with the fivefold partition of the reference results already discussed. Thus, for example, in the case of considering weight as the parameter under analysis, two complementary tests were carried out. In the first one, the system is trained with the *M* subjects with the lowest weight (where *M* is computed to the integer closest to 80% of the total number of participants in the datasets) while tested with the other 20% (those with the highest weight). Conversely, in the second test, the selection criterion is reversed and we considered the 80% with the highest weight for training and the rest for testing. In any case, very similar results (not presented here) were reached when the system was examined by separating users based on a fixed ‘hard’ threshold (for example, a certain value of the body weight).

In the case of a binary separation of the patterns based on the subject’s gender, two analyses were considered: when the classifier is trained with patterns exclusively generated by men and evaluated with movements performed by women (and vice versa). For these experiments, the size of each subset obviously depended on the percentage of male and female participants involved in each dataset. Thus, the 80/20 rule was not kept.

For comparison purposes, we also included in our analysis the outcomes of the detector for the case in which the partition of the subjects into the training and testing groups are performed at random, without taking into consideration any specific individual characteristic. By doing so, we try to determine whether the possible disparities with the baseline reference (under a ‘fair’ distribution) are simply due to the fact that the subjects in both groups are different, so that the detector, during the learning phase, tends to overfit certain particularities of the mobility of the training subjects, which are not necessarily associated with the physical characteristics that are analyzed here. For this ‘random’ distribution of users among the subsets, a fivefold cross-validation was again applied.

The results of the analysis for the five characteristics are presented in Tables 4, 5, 6, 7, 8. The main goal is to evaluate if the behavior of a classifier, which apparently performs accurately when a ‘fair’ distribution of participants for training and testing is considered, degrades if a different distribution of the samples of the individuals in the train and test subsets is applied. Thus, although all the possible combinations of pattern discrimination and classifying models were evaluated, for ease of comparison the tables only show (for each dataset) the performance metrics corresponding to the input feature set, algorithm and hyperparameters that yielded the best results for the ‘fair’ case (this baseline case, extracted from Table 3, is indicated in bold). Anyhow, from the massive tests executed with the other combinations, we can state that the conclusions achieved with the best performing classifier can be extrapolated in general to the behavior of the other algorithms.

The analysis of the performance of the classifiers, when the criterion to separate the training and test groups is based on weight (Table 4) and height (Table 5), seems to recommend to train the system with the tallest and most corpulent individuals. Except for the case of the UMAFall database, with the rest of the repositories it is verified that the detection algorithm generates better results when it is trained with individuals with greater weight and height (and tested with the thinnest and shortest subjects) than when the reverse operation is carried out. This behavior could be explained by the fact that thicker and taller subjects provoke more recognizable mobility patterns (e.g. higher acceleration peaks caused by impact), which may ease the discrimination between falls of ADLs. On the other hand, training the system with samples of thinner individuals can lead to a certain lack of references during the test phase when conventional ADLs are executed by more corpulent subjects with higher energy.

**Table 4:** Performance metrics of the best performing classifier (‘fair’ case) when the **weight** is used as a criterion to select the subjects of the training subset.

Dataset	Features and algorithm	Subjects included in the training subset	Se (%)	Sp (%)	$\sqrt{Se\cdot Sp}$ (%)
DOFDA	HCTSA features Naive Bayes (Gaussian)	Random selection of users	97.38	100.00	98.67
		**All (fair distribution)**	**97.37**	**100.00**	**98.67**
		Subjects (80%) with highest weight	94.81	100.00	97.37
		Subjects (80%) with lowest weight	93.51	100.00	96.70
Erciyes	Own selection of features SVM (quadratic kernel)	Subjects (80%) with highest weight	100.00	100.00	100.00
		**All (fair distribution)**	**99.62**	**99.18**	**99.40**
		Subjects (80%) with lowest weight	99.41	97.06	98.23
		Random selection of users	97.83	98.43	98.12
SisFall	HCTSA features SVM (cubic kernel)	Subjects (80%) with highest weight	100.00	100.00	100.00
		**All (fair distribution)**	**99.78**	**99.96**	**99.87**
		Random selection of users	99.74	99.96	99.85
		Subjects (80%) with lowest weight	85.33	100.00	92.38
UMAFall	Own selection of features KNN (Euclidean. 10 neighbors)	**All (fair distribution)**	**98.93**	**98.73**	**98.83**
		Subjects (80%) with lowest weight	100.00	95.38	97.67
		Random selection of users	98.28	97.05	97.66
		Subjects (80%) with highest weight	91.55	98.68	95.05
UP-Fall	Own selection of features SVM (linear kernel)	Subjects (80%) with highest weight	100.00	100.00	100.00
		**All (fair distribution)**	**99.59**	**98.02**	**98.80**
		Subjects (80%) with lowest weight	100.00	97.56	98.77
		Random selection of users	99.65	97.56	98.60

**Table 5:** Performance metrics of the best performing classifier (‘fair’ case) when the **height** is used as a criterion to select the subjects of the training subset.

Dataset	Features and algorithm	Subjects included in the training subset	Se (%)	Sp (%)	$\sqrt{Se\cdot Sp}$ (%)
DOFDA	HCTSA features Naive Bayes (Gaussian)	Tallest subjects (80%)	98.65	100.00	99.32
		Random selection of users	97.38	100.00	98.67
		**All (fair distribution)**	**97.37**	**100.00**	**98.67**
		Shortest subjects (80%)	93.59	100.00	96.74
Erciyes	Own selection of features SVM (quadratic kernel)	Tallest subjects (80%)	100.00	100.00	100.00
		**All (fair distribution)**	**99.62**	**99.18**	**99.40**
		Random selection of users	97.83	98.43	98.12
		Shortest subjects (80%)	91.22	93.73	92.46
SisFall	HCTSA features SVM (cubic kernel)	Tallest subjects (80%)	100.00	100.00	100.00
		**All (fair distribution)**	**99.78**	**99.96**	**99.87**
		Random selection of users	99.74	99.96	99.85
		Shortest subjects (80%)	99.78	99.83	99.80
UMAFall	Own selection of features KNN (Euclidean. 10 neighbors)	**All (fair distribution)**	**98.93**	**98.73**	**98.83**
		Shortest subjects (80%)	100.00	96.05	98.01
		Random selection of users	98.28	97.05	97.66
		Tallest subjects (80%)	77.78	96.67	86.71
UP-Fall	Own selection of features SVM (linear kernel)	Tallest subjects (80%)	100.00	97.83	98.91
		Shortest subjects (80%)	100.00	97.67	98.83
		**All (fair distribution)**	**99.59**	**98.02**	**98.80**
		Random selection of users	99.65	97.56	98.60

This difference in the performance of the FDS when the grouping of subject relies on the weight or height is not so evident when the body mass index (BMI) is used as the division criterion, as can be seen in Table 6. The BMI, which does not inform about the absolute values of weight and height but on the ratio between both parameters, seems to have a lower representativeness when characterizing the groups of individuals during falls and ADLs.

**Table 6:** Performance metrics of the best performing classifier (‘fair’ case) when the **Body Mass Index** (BMI) is used as a criterion to select the subjects of the training subset.

Dataset	Features and algorithm	Subjects included in the training subset	Se (%)	Sp (%)	$\sqrt{Se\cdot Sp}$ (%)
DOFDA	HCTSA features Naive Bayes (Gaussian)	Subjects (80%) with lowest BMI	100.00	100.00	100.00
		Random selection of users	97.38	100.00	98.67
		**All (fair distribution)**	**97.37**	**100.00**	**98.67**
		Subjects (80%) with highest BMI	94.81	100.00	97.37
Erciyes	Own selection of features SVM (quadratic kernel)	**All (fair distribution)**	**99.62**	**99.18**	**99.40**
		Random selection of users	97.83	98.43	98.12
		Subjects (80%) with lowest BMI	98.75	100.00	99.37
		Subjects (80%) with highest BMI	99.36	100.00	99.68
SisFall	HCTSA features SVM (cubic kernel)	**All (fair distribution)**	**99.78**	**99.96**	**99.87**
		Random selection of users	99.74	99.96	99.85
		Subjects (80%) with highest BMI	99.83	99.68	99.76
		Subjects (80%) with lowest BMI	98.22	99.62	98.92
UMAFall	Own selection of features KNN (Euclidean. 10 neighbors)	**All (fair distribution)**	**98.93**	**98.73**	**98.83**
		Subjects (80%) with lowest BMI	100.00	95.77	97.86
		Random selection of users	98.28	97.05	97.66
		Subjects (80%) with highest BMI	94.38	98.25	96.29
UP-Fall	Own selection of features SVM (linear kernel)	Subjects (80%) with highest BMI	100.00	100.00	100.00
		Subjects (80%) with lowest BMI	100.00	97.62	98.80
		**All (fair distribution)**	**99.59**	**98.02**	**98.80**
		Random selection of users	99.65	97.56	98.60

**Table 7:** Performance metrics of the best performing classifier (‘fair’ case) when the **age** is used as a criterion to select the subjects of the training subset.

Dataset	Features and algorithm	Subjects included in the training subset	Se (%)	Sp (%)	$\sqrt{Se\cdot Sp}$
DOFDA	HCTSA features Naive Bayes (Gaussian)	Youngest subjects (80%)	100.00%	100.00	100.00%
		Oldest subjects (80%)	100.00%	100.00	100.00%
		Random selection of users	97.38%	100.00	98.67%
		**All (fair distribution)**	**97.37%**	**100.00**	**98.67%**
Erciyes	Own selection of features SVM (quadratic kernel)	**All (fair distribution)**	**99.62%**	**99.18**	**99.40%**
		Youngest subjects (80%)	98.74%	100.00	99.37%
		Oldest subjects (80%)	98.07%	100.00	99.03%
		Random selection of users	97.83%	98.43	98.12%
SisFall	HCTSA features SVM (cubic kernel)	Youngest subjects (80%)	n.c.	99.56	n.c.
		Oldest subjects (80%)	100.00%	100.00	100.00%
		**All (fair distribution)**	**99.78%**	**99.96**	**99.87%**
		Random selection of users	99.74%	99.96	99.85%
		Subjects older than 50	98.03%	98.66	98.34%
		Subjects younger than 50	30.67%	99.32	55.19%
UMAFall	Own selection of features KNN (Euclidean. 10 neighbors)	Youngest subjects (80%)	n.c.	100.00	n.c.
		Oldest subjects (80%)	100.00%	100.00	100.00%
		**All (fair distribution)**	**98.93%**	**98.73**	**98.83%**
		Random selection of users	98.28%	97.05	97.66%
UP-Fall	Own selection of features SVM (linear kernel)	Oldest subjects (80%)	100.00%	97.67	98.83%
		**All (fair distribution)**	**99.59%**	**98.02**	**98.80%**
		Random selection of users	99.65%	97.56	98.60%
		Youngest subjects (80%)	97.62%	97.83	97.72%

*n.c.* not computable.

**Table 8:** Performance metrics of the best performing classifier (‘fair’ case) when the **gender** is used as a criterion to select the subjects of the training subset.

Dataset	Features and algorithm	Subjects included in the training subset	Se (%)	Sp (%)	$\sqrt{Se\cdot Sp}$ (%)
DOFDA	HCTSA features Naive Bayes (Gaussian)	Male subjects (testing with females)	100.00	100.00	100.00
		Random selection of users	97.38	100.00	98.67
		**All (fair distribution)**	**97.37**	**100.00**	**98.67**
		Female subjects (testing with males)	95.26	100.00	97.60
Erciyes	Own selection of features SVM (quadratic kernel)	**All (fair distribution)**	**99.62**	**99.18**	**99.40**
		Random selection of users	97.83	98.43	98.12
		Female subjects (testing with males)	97.29	98.06	97.68
		Male subjects (testing with females)	96.53	98.15	97.34
SisFall	HCTSA features SVM (cubic kernel)	Male subjects (testing with females)	100.00	99.92	99.96
		**All (fair distribution)**	**99.78**	**99.96**	**99.87**
		Random selection of users	99.74	99.96	99.85
		Female subjects (testing with males)	99.00	99.85	99.42
UMAFall	Own selection of features KNN (Euclidean. 10 neighbors)	**All (fair distribution)**	**98.93**	**98.73**	**98.83**
		Random selection of users	98.28	97.05	97.66
		Male subjects (testing with females)	95.35	98.02	96.68
		Female subjects (testing with males)	97.93	91.81	94.82
UP-Fall	Own selection of features SVM (linear kernel)	**All (fair distribution)**	**99.59**	**98.02**	**98.80**
		Male subjects (testing with females)	99.10	98.31	98.70
		Random selection of users	99.65	97.56	98.60
		Female subjects (testing with males)	98.51	95.56	97.02

Regarding age (Table 7), the analysis is constrained by the fact that in almost all the datasets, there are no elderly volunteers or, when older people do participate, they do not perform falling movements for safety reasons. DOFDA, UP-Fall and Erciyes databases only include people between 18 and 29 years (see Table 2). This homogeneity of the age range prevents drawing any conclusions about any discrimination of the subjects based on age since the youngest and oldest participants have a very similar age. In fact, with those datasets, the behavior of the classifiers does not register relevant differences if age is used to separate the test and training groups. Contrariwise, UMAFall and SisFall repositories do include samples captured from older people (aged up to 68 and 75 years, respectively). In both cases, when the older subjects are used in the test phase, the sensitivity cannot be calculated (which is indicated by the abbreviation *n.c.* in Table 7) since there are no falls performed by that age group. SisFall incorporates falls emulated by people over 50. Thus, to partially alleviate the lack of samples of falls in older people, the experiment was specifically repeated with the SisFall database, grouping in the test and training sets those older and younger (respectively) than 50 years. The results, although very limited by the scarcity of samples, are very revealing. Although training with samples generated by young people and testing with patterns executed by older people can produce high specificity, the obtained sensitivity is very low (30.67%). This could evince the fact that falls in older people follow very different mobility patterns than in younger individuals. These preliminary results are of great importance as they clearly question the usefulness of evaluating FDSs with databases generated from falls measured from a group of volunteers entirely composed of young people.

On the other hand, Table 8 shows the effects of using gender to separate the subjects employed in the training and test subsets. From the results we observe that this division does not substantially affect the effectiveness of the classifiers. In four of the analyzed databases, training with patterns generated only by men seems to lead to slightly higher quality metrics than those attained when the classifier is only trained with patterns captured from women. These divergences could be explained by the evident correlation that gender has with weight and height.

In any case (and except for the aforementioned case of training with young people), it should be noted that, for most databases, the differences caused by the segregation of the experimental subjects are not particularly significant. In fact, in some tests, the separation of test and training subsets based on a certain subjects’ characteristic leads to better results than those caused by a random distribution of participants. This may be an indication that the corresponding physical characteristic is not necessarily a key element to determine the nature of the mobility patterns.

### Results for a distribution of samples based on the nature of the movements

In this section we will show that the typology of movements (in particular the ADLs) used in the training phase constitutes a much more relevant factor than the characteristics of the experimental users to determine the extrapolation capacity of the detectors. For this purpose, we evaluated the classifiers when they are tested with types of movements different from those included in the training phase.

Given the enormous heterogeneity of ADLs (58 types) of the used databases, we propose to divide them into four groups or sub-categories, as also suggested in^6^, depending on the degree of physical effort required. Thus, we differentiate between:

1. Basic ordinary movements. This category encompasses those low-energy elementary routines that can be addressed by almost any subject. So, in the case of the five databases under analysis, this group would include activities such as: standing, lying, rising/descending from/to lying or kneeling, descending to sitting/rising from sitting or bending, and elementary hand movements (making a call, applauding).
2. Standard movements, which involve more physically demanding activities that require a higher degree of mobility, such as walking, going down, climbing stairs (up and/or down), or picking and object from the floor.
3. Sporting activities. In the datasets, this group covers up to four different actions: running, jogging, jumping and hopping.
4. Near falls. This category comprises those movements in which the experimental subject emulates a movement on the verge of losing his/her balance. In the five studied repositories, only Erciyes database has a certain number of these movements (including trips and stumbles).

In the case of falls, all the datasets incorporate very similar types, mainly distinguishable by the direction of the movements (lateral, backwards, frontal or vertical collapse). In some datasets, several variants are considered depending on the initial position of the subject or the emulated accident that causes the fall (slip/trip). For a more detailed description and comparison of the datasets refer to the study in^6^.

Table 9 shows the results of the performance of the algorithms that exhibit the best metrics when all the movement samples of a subcategory are excluded from the training patterns and exclusively used in the test phase. In all the combinations, a fair distribution of the subjects is employed between the training and test patterns (i.e. it is guaranteed that the detectors are trained with samples from all subjects). As in the previous section, the result of the fair case (in bold), in which the training patterns incorporate activities from all the categories, is used as the baseline reference.

**Table 9:** Performance metrics of the best performing classifier (‘fair’ case) when **different categories** of ADL are used in the training and the testing subsets.

Dataset	Features and algorithm	ADL categories used for		Se (%)	Sp (%)	$\sqrt{Se\cdot Sp}$ (%)
		Training	Test			
DOFDA	HCTSA features Naïve Bayes (Gaussian)	**All (fair distribution)**	**All (fair distribution)**	**97.37**	**100.00**	**98.67**
		Basic ADLs	Standard ADLs	100.00	68.00	82.46
		Standard ADLs	Basic ADLs	100.00	50.00	70.71
Erciyes	Own selection of features SVM (quadratic kernel)	**All (fair distribution)**	**All (fair distribution)**	**99.62**	**99.18**	**99.40**
		All but standard ADLs	Standard ADLs	99.34	98.90	99.12
		All but basic ADLs	Basic ADLs	99.34	97.22	98.28
		All but sporting ADLs	Sporting ADLs	98.68	95.65	97.15
		All but ‘Near Falls’	Near Falls	100.00	92.39	96.12
SisFall	HCTSA SVM (cubic kernel)	**All (fair distribution)**	**All (fair distribution)**	**99.78**	**99.96**	**99.87**
		All but basic ADLs	Basic ADLs	99.83	92.96	96.34
		All but sporting ADLs	Sporting ADLs	99.67	84.46	91.75
		All but standard ADLs	Standard ADLs	99.67	72.34	84.91
UMAFall	Own selection of features KNN (Euclidean. 10 neighbors)	**All (fair distribution)**	**All (fair distribution)**	**98.93**	**98.73**	**98.83**
		All but basic ADLs	Basic ADLs	100.00	93.84	96.87
		Standard ADLs	Standard ADLs	95.16	97.87	96.51
		All but sporting ADLs	Sporting ADLs	100.00	1.82	13.48
UP-Fall	Own selection of features SVM (linear kernel)	**All (fair distribution)**	**All (fair distribution)**	**99.59**	**98.02**	**98.80**
		All but basic ADLs	Basic ADLs	100.00	100.00	100.00
		Standard ADLs	Standard ADLs	98.77	95.93	97.34
		All but sporting ADLs	Sporting ADLs	100.00	2.17	14.74

The results clearly indicate the importance of diversifying the typology of the movements used for training, especially those that involve a greater degree of mobility. Thus, it is observed that classifiers do not tend to lose effectiveness when recognizing basic movements as ADLs, although they have not been used during the learning phase. On the contrary, performance (especially specificity) can dramatically degrade if movements of greater physical effort (especially sporting activities, which are mistakenly identified as falls) are considered in the test phase but not during training. Note, for example, that, in the case of the UMAFall and UP-Fall datasets, the specificity drops to less than 3% when a system trained with only basic and standard movements is evaluated with sport-like activities. This undoubtedly highlights the difficulties of the ML detectors to discriminate falls from other high-energy movements if they are not utilized to train the model.

## Conclusions

Wearable fall detection systems (FDSs) provide a cost-effective and non-invasive method to automatically discriminate falls from other activities of daily life basing on inertial signals (mainly accelerometry). The most effective wearable fall detection systems in the literature are founded on machine learning algorithms that usually require a learning phase. This training is usually carried out on movement patterns (emulated falls and ADLs) captured from a set of experimental subjects different from the target public of these systems, mainly older adults, who, for obvious reasons, cannot emulate falls for a final fine-tuning of the classifiers.

However, in the vast majority of studies in the literature, FDSs are evaluated with the same subjects who generated the training samples. This study has focused on systematically analyzing the performance of a ML-based FDS when the characteristics of the individuals used in the test are different from those participants employed for the training. By using five well-known public datasets, four ML algorithms (with different hyperparameter configuration) and two alternatives to select the input feature sets, we investigated the impact of this separation of training and testing subjects on the effectiveness of the detectors. In particular, we examined the discrimination capability of the classifiers when the training and test subjects are separated based on five criteria: weight, height, body mass index, gender and age. In all cases, the performance metrics of the classifiers (sensitivity, specificity and geometric mean of both parameters) were compared with those obtained for: (1) the (optimal) case in which the test and training individuals coincide, (2) the case in which the separation of the subjects in both groups (test and training) is randomly executed. The results indicate that this segregation of the experimental subjects can cause some losses in the classification process. For most of the used datasets, the underperformance seems to be somewhat lower if the tallest and heaviest individuals are not included in the training group, which could be justified by the fact that corpulent persons generate confusing acceleration patterns (with larger peaks) when they perform ADLs. The impact of gender (if we discount its possible correlation with weight or height) or BMI is shown to be of secondary importance. Regarding age, the preliminary results illustrate the difficulty of applying detection algorithms to older persons when they have been trained exclusively with young subjects. In any event, any conclusion derived from the split of the train and test groups based on an age criterion should be taken with great caution since the study is strongly limited by the lack of fall patterns associated with elderly volunteers. Just one dataset (SisFall) incorporates falls of subjects over 50 years old. In addition, the fact that employed falls are actually emulated may also distort the registered divergences between the two groups (younger and older participants), as they may be really caused by the differences in the way these two groups emulate a fall accident and not by the compensatory movements that they would perform during an actual fall.

In any case, the study discloses that the characteristics of the participants have much less impact on the effectiveness of the classifiers than the type of movements used for training and testing. Moreover, the performed tests show that the algorithms clearly tend to overlearn the particular ADLs used for training, in such a way that, during the test phase, they have difficulties to identify new ADL routines, especially if they involve sudden or highly energetic movements (such as sport-type activities), as they are misclassified as falls. These results show the importance of training the fall detectors with as many activities as possible. In contrast with the procedure typically employed by the related literature, the discrimination and extrapolation capability of FDSs should be equally tested against a wide variety of ADLs and falls, including movements not present in the training set.
